# The Nutritional and Health Benefits of Kiwiberry (*Actinidia arguta*) – a Review

**DOI:** 10.1007/s11130-017-0637-y

**Published:** 2017-10-07

**Authors:** Piotr Latocha

**Affiliations:** Department of Environmental Protection, Faculty of Horticulture, Biotechnology and Landscape Architecture, Warsaw University of Life Sciences – SGGW, Nowoursynowska 159, 02-776 Warsaw, Poland

**Keywords:** Hardy kiwifruit, Chemical composition, Antioxidant activity, Allergy, Superfood, Functional food

## Abstract

The kiwiberry (*Actinidia arguta*) is a new product on the market that is enjoying growing consumer acceptance around the world. This widespread interest has created increased demand for identification of the kiwiberry’s nutritional health benefits. Containing over 20 essential nutrients and a range of vitamins, the kiwiberry comes near the top of fruits classed as superfoods. It is one of the richest sources of vitamin C with up to 430 mg/100 g fresh weight (FW) and is considered the richest dietary source of *myo*-inositol (up to 982 mg/100 g FW). The kiwiberry is also one of the richest sources of lutein (up to 0.93 mg/100 g FW) in commonly consumed fruit. Furthermore, containing up to 1301.1 mg/100 g FW phenolics and significant amounts of the essential minerals of potassium, calcium and zinc, the kiwiberry rates very highly as a ‘healthy food’. The type and number of this fruit’s medicinally promising nutrients have motivated ongoing investigations into its antioxidant, anti-tumour and anti-inflammatory properties. Early research has pointed to the kiwiberry being a very promising treatment for some cancers and health issues involving the gastrointestinal system, hypercholesterolemia and certain cancers. A pharmaceutical composition of *A. arguta*, *A. kolomikta*, and *A. polygama* extracts has already been registered for the prevention and treatment of some immune (inflammatory) mediated diseases, as well as the treatment of some non-allergic inflammatory diseases. This paper reviews and highlights the limited nutritional and therapeutic information currently available on the kiwiberry, a minor fruit possessing such major properties.

**Electronic supplementary material:**

The online version of this article (10.1007/s11130-017-0637-y) contains supplementary material, which is available to authorized users.

## Introduction

Human diets rich in fruit and vegetables are considered to be the healthiest. Novel fruits and berries are increasingly being introduced onto the local and world food stage. It is commonly accepted that these fruits are an important and healthy source of biologically active substances, including vitamins, phenolics, carotenoids and minerals, and that they promote quality of life [1, 2]. In recent years, there has been growing interest in lesser known fruits such as cornelian cherry, honeysuckle, hawthorn, chokeberry, rowanberry, elderberry, sea buckthorn, medlar, elder, bilberry and kiwiberry, all fruits that are unusually rich in health-promoting compounds. Some of them can be consumed fresh while others are eaten after processing [3, 4]. The main driver behind their introduction is increased demand for exotic tastes coupled with a unique and healthy eating experience.

Kiwifruit is the main representative of the *Actinidia* genus. It is known worldwide and highly appreciated for its delicious taste and health-promoting properties [5]. The other edible and very promising *Actinidia* species is *A. arguta* (Siebold et Zucc.) Planch. ex Miq., also known as kiwiberry, hardy kiwi, baby kiwi or mini kiwi. This exotic species is very interesting and promising given the horticultural advantages it has over kiwifruit, especially its high frost hardiness (down to −30 °C in midwinter) and relatively short vegetation period. This capacity for the kiwiberry to grow in a shorter growing season in colder climates makes it an alternative to the warmer climate kiwifruits of New Zealand, potentially allowing it to become the first *Actinidia* to be commercially cultivated in colder north European countries [6]. The kiwiberry has been researched in some countries since the middle of the twentieth century, but its commercial production is relatively new and began on a small scale in the US and some European countries in the 1980s and 1990s. Currently the kiwiberry is commercially cultivated in several countries including the US, Chile, New Zealand, Australia, China and most European countries, *e.g.*, France, Belgium, Italy, the Netherlands, Switzerland, Austria, Poland and Germany [7, 8]. Being a vine, *Actinidia arguta* requires special support for growth and high-quality fruit production, Fig. 1 (online resource). Considered a niche product, the total worldwide kiwiberry crop is estimated to be about 1600 t *per* year. About half of this is produced in the US, while the second largest group of producers is SCAAP Kiwifruit France, the owner of the Nergi® brand, which produces more than 300 t *per* year. Another strong group of kiwiberry growers is connected to Ghent University in Belgium and it produces more than 200 t *per* year in several European countries. In New Zealand, kiwiberry cultivation is concentrated in the Bay of Plenty, with the annual crop estimated to be about 160 t. Generally, the world area of kiwiberry cultivation and the annual crop is slowly but steadily increasing. A spectacular increase has only been observed in China, where more than 1200 ha of new plantations have been recently established.

In contrast to the kiwifruit industry being dominated by one cultivar, Hayward, there are many different kiwiberry cultivars, although not all of them have the same commercial potential [9]. Patented cultivars such as Hortgem Tahi, Hortgem Rua or Takaka Green are commonly cultivated in New Zealand. Its fruit is predominantly green without any blush. In the US, the cultivar Ananasnaya, with a strong red blush, is the most extensively grown, but other selections, such as Passion Popers and Aloha Annas, are also cultivated. In Europe, two cultivars are commonly commercially cultivated: Weiki, which is very similar in appearance to Ananasnaya, and Geneva which has spherical green fruit and a weak reddish-brown blush. Finally, in China local cultivars such as Huan no. 1, Long into the 2nd or Quebec Green (entirely green) are the most popular.

*A. arguta* are small grape-sized fruit, with a thin, edible and predominantly green skin, but some red-coloured cultivars are also available – Fig. 1 (online resource). The fruit is very aromatic with a sweet, intense flavour that has been compared to blackcurrant, pineapple, ripe strawberry, pear, banana, melon and other tropical flavours [10] and can be used for eating fresh and/or for jam and wine.

In light of the growing consumer acceptance of kiwiberry fruit worldwide, there has been an increased focus on identifying the health benefits derived from its consumption. In recent years, the number of scientific publications related to *Actinidia arguta* has increased significantly. Current research by various scientific institutions points to kiwiberry, similar to kiwifruit, being one of the most nutritionally rich fruits in the world. It is considered to be nature’s perfect fruit because it is an excellent source of antioxidants. Most nutritional research conducted on the kiwiberry compares it to the more familiar kiwifruit. Unfortunately, however, there are currently no published scientific findings or comparisons.

Kiwifruit and papaya fruit are considered the most nutrient-dense fruits among commonly consumed fruits and are usually referred to as “healthy fruit” [5]. The concentration of such a large number of different compounds in a single fruit is rare. A great advantage of the kiwiberry (over kiwifruit) is its delicate, edible grape-like skin, which contains up to 15 times more antioxidants than the fruit pulp [11, 12]. In contrast to the kiwiberry, kiwifruit requires a tough, fuzzy, unpalatable skin to be peeled prior to eating.

The kiwiberry is considered an excellent source of vitamin C, vitamin B_8_ (*myo*-inositol), lutein, β-carotene, chlorophylls, enzyme actinidin and antioxidants, as well as dietary fibre. It is also a good source of vitamins A and E and some minerals (potassium, calcium, magnesium, copper, iron, manganese), while also containing meaningful amounts of vitamin B_6_ and other vitamins and minerals. Presently, many nutritional components of the kiwiberry remain unstudied and await further analysis.

Considerable published research describes the kiwiberry as having a similar chemical composition to kiwifruit, but containing significantly higher proportional amounts of particular compounds [13, 14]. Therefore, on a given weight basis, kiwiberry could have significantly greater health benefits. Demand for natural foods rich in biologically active compounds is ever increasing. The derived health benefits are proven to be more effective when these compounds occur together [15]. It should be pointed out that the chemical composition of the kiwiberry is not uniform and genetic variability, various aspects of cultivation, harvesting, storage and processing, as well as many environmental factors (*e.g*., soil fertility, water availability, temperature and insolation) may affect the chemical and nutritional properties of this fruit [16–18].

## Kiwiberry Nutrition

To address the complexities of changes in composition resulting from multiple factors, a large number of samples should typically be analysed using standardised methods. In the main, chemical analyses are conducted on fruit ripened to a “ready-to-eat” state to ensure that the data are reflective of what would normally be consumed. The average amounts of chemical compounds in kiwiberry versus kiwifruit are summarised in Table 1 (online resource).

### Basic Chemical Properties (Dry Matter, Soluble Sugar, Pectins, Organic Acids, Titratable Acidity, pH, Ash, Fibre and Protease)

Kiwiberry dry matter (DM) content depends on the cultivar and the growing conditions but, according to available research, varies between 14.6 and 25.5%, in contrast to Hayward kiwifruit having 15.3–17.2% DM [14, 16, 19–21]. Unlike kiwifruit, the kiwiberry is a good source of pectins. The amounts of pectin vary widely in kiwiberry cultivars and range from 2.17 to 3.30% [20]. Kiwiberry soluble solid content at eating maturity stage ranges between 12.1–16.8° Brix and is higher than that of Hayward kiwifruit (13.2–13.8° Brix) [19, 20]. Kiwifruit and kiwiberry soluble sugars and organic acid composition is not uniform and may influence fruit palatability [22]. Kiwifruit (*A. deliciosa*) and kiwiberry (*A. arguta*) have a similar total concentration of soluble sugars, containing about 8.1–8.8 and 3.9–9.6 g of sugars *per* 100 g of fresh weight (FW), respectively [20, 23–25]. The main soluble sugars in *A. deliciosa* fruits are glucose and fructose, with sucrose present in smaller amounts. In contrast, sucrose is the predominant sugar in *A. arguta* berries, followed by glucose and fructose [7, 26, 27].

The balance of sugar and organic acids in kiwifruit and kiwiberry contributes greatly to their sensory qualities [28]. In this regard, most kiwifruits and kiwiberries have citric and quinic acids as their predominant organic acids, followed by malic acid [7, 20, 29]. In contrast to other common fruits, the acid-tasting quinic acid predominates, but the proportion of quinic acid in the kiwiberry is lower than in kiwifruit [25]. According to Wojdyło et al. [20], kiwiberry organic acids, totalling 16.85–21.06 g/100 g FW, consist of citric acid (5.75–9.56 g/100 g FW), quinic acid (4.17–8.20 g/100 g FW), malic acid (1.37–4.34 g/100 g FW), oxalic acid (1.70–3.67 g/100 g FW), shikimic acid (0.03–0.23 g/100 g FW) and succinic acid (0–0.28 g/100 g FW). Citric acid is the dominant organic acid, accounting for 30–50% of the total organic acid content. Significantly divergent results have been produced in Japan by Nishiyama et al. [24]. The authors analysed various species of actinidia and determined the content of the three main organic acids (citric, quinic and malic acid) in *A. arguta* fruit to be 0.54–1.37, 0.51–0.73 and 0.10–0.30 g/100 g FW, respectively. In contrast to the kiwiberry, Hayward kiwifruit contains only 2.19–2.41 g organic acids in total *per* 100 g FW [24]. The kiwiberry, with a pH range of 3.1 to 3.6, and kiwifruit, pH 3.1 to 4.0, are both classified as “high acid” foods. Their ash content ranges between 0.62 to 0.94% for kiwiberry and is 0.61% for the kiwifruit Hayward [14, 20].

Kiwiberry fruit contains about 2.0–3.8% TDF and appreciable amounts of proteases. Kiwiberry TDF, expressed as a percentage of FW is similar or higher than kiwifruit, with insoluble fibre and soluble fibre of 2.1–3.1 and 0.8–1.0%, respectively. The principal protease is cysteine protease actinidin, which acts similarly to the better-known protease papain [5]. Significant differences in actinidin concentration exist among kiwiberry and kiwifruit species and cultivars. Generally, the amounts of actinidin in the three main species (*A. deliciosa*, *A. chinensis* and *A. arguta*) vary at 2.9–4.4, 0–5.7 and 1.6–10.7 mg/mL of fruit juice, respectively [30]. The protease activity measured for different kiwiberry cultivars varies appreciably from 25.7 to 114.0 nM *p*NA/min and is much higher than for kiwifruit (6.3–10.1 nM *p*NA/min) [30].

### Vitamins

Kiwifruit and kiwiberry are rich in various vitamins. Most notably, *Actinidia* fruits are exceptionally high in ascorbic acid (vitamin C). Amounts may vary considerably between species and cultivars however. The vitamin C content of the three main species generally varies from 22.8 to 430 mg/100 g FW, with levels in *A. arguta* (kiwiberry) generally being somewhat higher than in *A. chinensis* and *A. deliciosa* (both kiwifruit) (on average 150 mg, 103 mg and 65 mg/100 g FW respectively) [31].

The most popular kiwiberry cultivars in Europe – Weiki, Ananasnaya and Geneva – differ in vitamin C content between 45.4 and 107.7 mg/100 g FW respectively [17]. Some hybrids, *e.g*., Issai, are especially rich in this vitamin, containing up to 222 mg vitamin C/100 g FW. Unfortunately, the cultural morphological features of ‘Issai’ limit this hybrid to more non-commercial applications. Some new Chinese kiwiberry cultivars such as Kui Lu can contain up to 430 mg vitamin C *per* 100 g FW [32]. The highest amount of vitamin C has been found in the kiwiberry *Actinidia kolomikta*, which accumulates up to 1500 mg/100 g FW [19, 33].

Vitamin C is relatively unstable because it is easily oxidised in the presence of oxygen and light and heavy metal ions. However, the kiwiberry and even kiwifruit with its longer shelf life manage to retain their vitamin C levels well, displaying only a slight reduction over time [7, 34].

In addition to vitamin C, *Actinidia* berries are a good source of several other vitamins. They contain significant amounts of vitamin B-complex, mainly B_1_ (0.01–0.05 mg), B_2_ (0.02–0.11 mg), B_3_ (0.50–1.55 mg), B_5_ (3.80–5.60 mg), B_6_ (1.10–1.90 mg) and B_8_ (266–982 mg) in 100 g FW [24, 35–37]. Unfortunately a literature search failed to locate scientific research on vitamin B_9_ (folic acid) in the kiwiberry analysis. According to Nishiyama et al. [24], the kiwiberry has levels of *myo*-inositol (vitamin B_8_) four to six times higher than those of kiwifruit (up to 982 mg and 153 mg/100 g FW respectively). This makes the kiwiberry one of the richest dietary sources of the hexahydric sugar alcohol, an important vitamin B-complex component. *A. arguta* fruit has almost the highest *myo*-inositol content of all foods. In some cases *myo*-inositol may constitute roughly 60% of all sugars contained in actinidia fruits [38]. The kiwiberry is an important source of vitamin E (4.6–5.3 mg/100 g FW) which, being a fat-soluble vitamin, is believed to be contained predominantly in the seeds. In such a form, this vitamin would not be bioavailable as the seeds typically resist digestion [5]. However, more recent analysis has shown that the main α-tocopherol form of vitamin E is in the flesh, possibly associated with cell membranes, and therefore potentially bioavailable [39]. Unfortunately, to date there are no scientific reports on a vitamin K analysis of the kiwiberry. Data on the vitamin A content of kiwiberry is sourced from Korea. According to an analysis undertaken at Suwon Women’s University Food Analysis Centre, vitamin A calculated as retinol activity equivalents from different kiwiberry cultivars varied from 37.3 to 84.5 μg RAE/100 g FW [40].

### Phenolic Compounds

Both kiwifruit and the kiwiberry are fairly significant sources of phenolics, which are commonly considered to be very effective antioxidants. The reported TPC may vary depending on various factors, including the plant’s growing conditions and the laboratory’s extraction and determination method. The content of *Actinidia arguta* polyphenols was found to be up to more than three times that of Hayward kiwifruit [41]. In kiwifruit the TPC recalculated as GAE was 41.7–267 mg *per* 100 g FW [17, 37, 41, 42]. Park et al. [42] suggest that the main group of phenolics in kiwifruit are flavonoids, tannins and flavanols. By contrast, the kiwiberry TPC assayed much higher. According to An et al. [43], the kiwiberry contains 118.2–191.6 mg GAE/100 g FW phenolics, including 28.8–40.4 mg CE/100 g FW flavonoids. Other research has reported the kiwiberry as having phenolics of up to 426 mg as GAE/100 g FW [19, 44, 45], but other studies have reported that the kiwiberry TPC calculated as a sum of all identified compounds ranges from 443.2 to 1301.1 mg/100 g FW and is significantly cultivar dependant [20]. The authors identified flavan-3-ols (based on the UPLC-PDA method) as the major class of polyphenols in kiwiberry fruits, representing between 96 and 99% of total polyphenolic compounds. Flavonols are the second major class of phenolic compounds (1–4%), followed by phenolic acids (0.4–0.7%) and finally anthocyanins (0–0.8%). Based on reference standards or mass spectra, the authors identified about 24 different compounds, twenty-two of which were identified for the first time in the fruits of *Actinidia*. The largest group was flavonols (16 compounds), mainly quercetin and kaempferol derivatives, followed by phenolic acids (seven compounds). Other sources report the kiwiberry to contain gallic acid, chlorogenoc acid, tannic acid, 2,4-dihydroxybenzoic acid, caffeic acid, (+)-catechin, (−)-epicatechin, rutin and quercetin [11, 19]. As the phenolics protect plant organisms against harmful environmental factors, they are found concentrated in the skin/peel of a fruit. With the kiwiberry, the skin or peel is associated with phenolic concentrations that are much higher than in the pulp [12]. The higher amount of phenolics may possibly reflect exposure levels to various environmental stress factors.

### Pigments

Depending on the species and cultivar, *Actinidia* fruit also contain substantial amounts of different pigments, including carotenoids, chlorophylls, and anthocyanins. Lutein and β-carotene, both potent antioxidants, are the most concentrated carotenoids of *Actinidia* fruit. Among commercial *Actinidia* species, the kiwiberry contains the highest levels of lutein and β-carotene, up to 0.93 and 0.29 mg/100 g FW, respectively [46]. Occurring in smaller amounts are zeaxanthin (0.02–0.04 mg/100 g FW), violaxanthin (0.01–0.12 mg/100 g FW) and trace amounts of α-carotene [17, 41]. Significant quantitative differences in these compounds reported in Europe and Japan suggest a significant influence of climate and genetic make-up on fruit pigment composition.

Some red fleshed kiwiberry species and cultivars (*e.g*., *A. melanandra* and *A. arguta* or its hybrids) also contain small amounts of anthocyanins. Available research indicates that the total anthocyanin content was the highest in *A. melanandra* (50.4–98.5 μg/100 g FW) and red-fleshed *A. arguta* cultivars (161.2–206.1 μg/100 g FW), where anthocyanins are present in the skin, pericarp and core of the fruits [47, 48]. Other research confirms the presence of anthocyanins in some green-fleshed kiwiberry cultivars as well (up to 129.8 μg /g DW) [20, 41]. Recent research identifies cyanidin-3-*O*-sambubioside as the major anthocyanin in the Ken’s Red cultivar of kiwiberry [20].

Another pigment closely associated with the fruit of *Actinidia* is chlorophyll. The kiwiberry, containing between 2.6 and 4.2 mg/100 g FW, has up to 3.2 times the chlorophyll concentration of kiwifruit (1.3–2.7 mg/100 g FW) [41, 46]. Chlorophyll *a* is the predominant form in both kiwifruit and kiwiberry [17, 46].

### Minerals

The mineral composition of the kiwiberry is quite variable and depends on its genetic features (cultivar) and growing conditions such as soil and weather. The order of the relative quantity of macroelements found in kiwiberry is K > Ca > P > Mg > Na, and of microelements is Fe > Zn > B > Mn > Cu. On the basis of available research, the kiwiberry is a good source of potassium, calcium and magnesium [16, 37, 49–51]. Depending on the cultivar and year of cultivation, the kiwiberry may contain 162.7–382 mg K/100 g FW, 51.5–120.1 mg Ca/100 g FW, 31.7–80.2 mg P/100 g FW, 10.0–23.2 mg Mg/100 g FW, and just 1.2–9.6 mg sodium [16]. The concentrations of potassium and magnesium in the kiwiberry are similar to the levels found in Hayward kiwifruit, while kiwiberry calcium concentration is approximately twice that of kiwifruit [14, 52].

The kiwiberry is also a significant source of microelements, containing 0.31–1.15 mg Fe/100 g FW, 0.18–1.45 mg Zn/100 g FW, 0.18–0.48 mg B/100 g FW, 0.03–0.24 mg Mn/100 g FW and 0.05–0.16 mg Cu/100 g FW. These values usually make the kiwiberry a richer or similar source of these minerals than kiwifruit [16, 50]. The same proportion of macro- and microelements in different kiwifruit cultivars tested in Iran has been found by Samadi-Maybodi and Shariat [53] and the kiwiberry Mitsu-ko tested in Japan [7]. According to Japanese studies, the content of K, Ca, Mg, Mn and Zn in *A. arguta* fruit is higher than in Hayward kiwifruit. Similar results for *A. arguta* have been obtained by Martens [49] and for *A. arguta* and *A. purpurea* hybrids by Bieniek [50]. The consistent correlation between micronutrient proportional concentrations of fruits of the *Actinidia* genus suggests that this is dependent more on its genetic make-up than on growing conditions.

### Amino and Fatty Acids

The kiwiberry also contains amino acids. According to research undertaken by Jin et al. [37], the content of total amino acids in the kiwiberry is 601–1220 mg/100 g FW, of which 199–414 mg are essential amino acids. The authors identify about 20 different amino acids in the kiwiberry, with the two major amino acids being glutamic acid and aspartic acid - Table 1 (online resource).

The lipid content of the kiwiberry is similar to Hayward kiwifruit and is restricted to the seeds almost entirely, with a small level of membrane-associated lipids located within the flesh [14]. The defined fatty acids include 13.9–30.5% saturated fatty acids and 70.4–85.8% unsaturated fatty acids. The analysis of fatty acids shows that the major fatty acids in the three kiwiberry cultivars are palmitic acid as a saturated fatty acid and α-linoleic acid as an unsaturated fatty acid [37]. This makes its oil of interest for nutritional uses.

### Antioxidant Activity

Given the type and number of this fruit’s medicinally promising nutrients, there are ongoing investigations into its antioxidant and anti-inflammatory properties that might help protect against cardiovascular diseases, cancer and other degenerative disorders. Many of the abovementioned bioactive compounds possess free radical scavenging abilities. The kiwiberry AA of scavenging free radicals in the body could help reduce certain diseases thought to be modulated by free radicals [43]. Kiwiberry extracts show stronger antioxidant activity than kiwifruit, as measured by different methods related to different groups of compounds. Just as the kiwiberry’s chemical composition differs depending on various genetic and environmental factors, its ability to scavenge free radicals is also variable. Unfortunately, different measurement methods and extraction procedures (the solvents used, the duration and the temperature of extraction) can make these results difficult to compare. The average AA of different extracts from the kiwiberry versus kiwifruit is summarised in Table 2 (online resource).

According to Latocha et al. [17], the AA of kiwiberry hydrophilic extracts based on the ABTS assay ranges from 1.73 to 4.22 mg AAE/g FW on average and is similar to that of kiwifruit (1.83–2.06 mg AAE/g FW). These results are comparable to values obtained by An et al. [43] for the kiwiberry in South Korea (1.10–2.10 mg AAE/g FW). Meanwhile Leontowicz et al. [41], using the same assay and solvent, indicate a much higher AA of the kiwiberry (47.2–122.4 μM TE/g DW) compared to kiwifruit (13.5–16.1 μM TE/g DW). In contrast, the AA of lipophilic extracts based on the ABTS assay range from 10.8–29.9 and 11.1–13.5 μM TE/g DW [41] or 0.93–2.64 and 0.95–1.36 mg AAE/g FW [17, 54, Latocha unpubl.] for kiwiberry and kiwifruit respectively, showing great similarity. The second result is comparable to that obtained for oranges (1.42 mg AAE/g FW), but lower than the one for strawberries (4.72 mg AAE/g FW) [54].

The kiwiberry AA measured by the DPPH assay indicates the same proportions as that obtained by the ABTS assay. Leontowicz et al. [41] maintains that both hydrophilic and lipophilic extracts of the kiwiberry (10.5–42.5 and 9.0–15.7 μM TE/g DW, respectively) show much higher AA than kiwifruit (6.6–7.9 and 6.0–6.9 μM TE/g DW, respectively). Other research work undertaken by Fisk et al. [44] in Oregon in the US, Latocha et al. [17] in Poland and An et al. [43] in South Korea, which used the same method for lipophilic kiwiberry extracts, show similar results - Table 2 (online resource). The research performed on selected fruits in Japan [45] show that the kiwiberry AA measured by hydrophilic ORAC assay (88.7–99.7 μM TE/g FW) is more than 13 times higher than that of kiwifruit (5.2–7.1 μM TE/g FW) and 30% higher than blueberry (65.6 μM TE/g FW). In turn, the AA of kiwiberry and kiwifruit lipophilic extracts indicate greater similarity (ORAC assay). Other experiments that used CUPRAC and FRAP assays and both types of solvent [41] confirmed that the kiwiberry has significantly higher AA than kiwifruit, irrespective of the assay used. The results of the kiwiberry AA against naturally occurring hydroxyl radicals (^•^OH), which is claimed to be the most reactive form in biology, confirm that kiwiberry extracts can reduce these radicals by up to 70.7% [17]. Confirmed by considerable additional research, kiwiberry antioxidant activity is strongly correlated with TPC and TAA content in fruits [16, 19, 41, 45, 55].

## Kiwiberry Health Benefits

Originating in China, *Actinidia* plants were historically used in folk medicine for the symptomatic relief of numerous disorders, including digestive problems, dyspepsia, rheumatism and haemorrhoids. Various Chinese cancer therapies purportedly also used *Actinidia*. The potential health and healing benefits connected with kiwifruit come from its rich nutritional composition and the fact that so many different compounds interact synergistically. This nutrient-dense superfood is a source of beneficial vitamins, minerals, and compounds that are essential for good health.

Kiwifruit’s therapeutic value in treating certain diseases has been identified and described in various literary sources. One of the best-known benefits of the *Actinidia* fruit is its support of the gastrointestinal system. Extract of *Actinidia* fruit has been shown to support the growth of good flora while suppressing harmful flora [56]. Its enzymatic tenderising property has also been shown to help in the digestion of meat in the digestive tract. Taken together, these supporting actions of kiwiberry and kiwifruit facilitate improved digestive tract health. Some digestive health products based on kiwifruit extracts have therefore already been developed and marketed (*e.g*., Zylax in New Zealand or Kiwi-Klenz in Germany).

Currently there is little research published on the human health benefits of the kiwiberry. Most research to date has been carried out on animal models or *in vitro*. Pioneering research on the kiwiberry is being conducted in Poland to demonstrate any health benefits identified [57]. The authors have reported that *Actinidia* fruits demonstrate protective activity against induced hypercholesterolemia in rats. Two kiwiberry cultivars commonly cultivated in Europe – Geneva and Weiki – have a much stronger protective effect than the kiwifruit *A. deliciosa* Hayward or *A. eriantha* Bidan. The mechanism protecting against cholesterolemia involves changing the expression of genes connected with the metabolism of fat in the liver and aorta. Some *in vitro* research conducted in China and Korea confirms the high inhibitory effect of different kiwiberry extracts on some lines of human cancer cells such as HepG2 and HT29 [55, 58, 59] and Hep3B and HeLa *in vitro* [60]. Subsequent research by Yu et al. [59] has demonstrated some promising anti-cancer properties of *A. arguta*. Specifically, a polysaccharide of *A. arguta*, identified as AAP-3b, has inhibited human liver cell tumour proliferation *in vitro*. This inhibition is associated with the growth interruption of liver tumour cells as well as tumour cell death. This research suggests the future potential of healthy *A. arguta* fruit or constituents for use in anti-tumour treatment. Nishimura et al. [61] have investigated the inhibitory effect of *A. arguta* juice on the mutagenicity of food-derived carcinogens and polycyclic aromatic hydrocarbons. The authors suggest that the components in *A. arguta* responsible for the antimutagenicity are water-soluble, heat-labile phenolic compounds. These results suggest that components in *A. arguta* are promising candidates to be chemopreventive agents. Chinese research has shown that *A. arguta* crude alkaloids possess anti-fatigue effects and increase exercise performance in mice [62], therefore *A. arguta* could be considered as an anti-fatigue dietary supplement (nutraceutical) in the category of functional foods. Korean researchers have found that the ethyl acetate fraction from *A. arguta,* containing physiological phenolics, might enhance drug-induced amnesia through acetylcholinesterase inhibition and neuroprotection [63]. The latest interesting research on the kiwiberry by An et al. [43] investigates its anti-inflammatory effect using a lipopolysaccharide (LPS)-stimulated RAW 264.7 murine macrophage cell line. The authors conclude that kiwiberry extracts suppress the secretion of pro-inflammatory cytokines, including interleukin-6 and tumour necrosis factor-α, from LPS-stimulated RAW 264.7 cells, and can be considered a potential anti-inflammatory agent. Cellular-based measurements of antioxidant capacity show that the kiwiberry extracts have cellular antioxidant capabilities. Such cellular antioxidant effects are possibly directly attributed to their antioxidant properties or to the inhibition of free radical generation via their anti-inflammatory effects. Finally, after their mice-based kiwiberry research Gan et al. [35] conclude that kiwiberry juice can significantly activate SOD and GSH-Px in red blood cells and enhance liver health remarkably. It can also increase the antioxidative activity of enzymes and reduce peroxidation in ageing organisms, thus delaying the ageing process.

The aforementioned scientific studies provide new insight into the health benefits of the kiwiberry. Furthermore, the cited research demonstrates and advances the potential role of *Actinidia arguta* extracts in preventative health care.

## Kiwiberry Allergy

The allergenicity of kiwiberry constituents is neither well studied nor understood. Kiwifruit, more widely consumed than the kiwiberry, has been subject to an increasing number of reported allergies as its worldwide popularity grows. Two to 3 % of the human population display hypersensitivity to the actinidin contained in fruit, with the most common allergic reaction symptom being an oral dermatitis. Interestingly, individuals allergic to *A. deliciosa* kiwifruit demonstrated less severe symptoms following consumption of *A. chinensis* kiwifruit. It has been reported that some allergy symptoms can be influenced by other food components consumed along with kiwifruit [64]. Kiwifruit allergy is often cross-reactive with other allergies, such as that to birch and grass pollen. It also appears to have cross-reactivity to the latex sensitivity syndrome. Several constituents of *Actinidia* fruit have been tentatively identified as allergenic. Specifically, 11 kiwifruit allergens have been registered in the Allergen Nomenclature database. These include the 30-kd cysteine protease Act (Act d1), an enzyme that catalyses the hydrolysis of peptide bonds and is considered to be the main allergen in kiwifruit, 24-kd thaumatin-like protein (Act d2), 26-kd cell wall protein kiwellin (Act d5), and cysteine protease inhibitor cystatin (Act d4). However, recent concerns have expressed that some kiwifruit proteins may have been added prematurely to the database without adequate scientific rigour in assessing allergenicity or only based on potentially unreliable *in vitro* assays. IgE binding, rather than clinically relevant reactivity, is inappropriately used to confirm allergenicity [65]. Furthermore, some reports indicate that kiwiberry fruit can even protect against some allergy types. According to Park et al. [66], PG102 possesses orally active immune-modulating activity in mice. In research conducted by Park et al. [67, 68], Choi et al. [69] and Kim et al. [70], kiwiberry extracts have effectively reduced the levels of total IgE in treated atopic dermatitis in mice or rats. The same results have also been reported for dogs [71]. Moreover, Kim et al. [72] mention the anti-allergic effects of PG102 in a murine ovalbumin-induced asthma model.

Some clinical studies conducted with PG102 have effectively reduced the levels of total IgE in apparently asymptomatic subjects with atopy [73]. Furthermore, other researchers suggest that PG102 has great potential as a safe (with minimal risk of side effects) orally active immune modulator for the therapy of various human allergic diseases [66]. Research undertaken by Korean scientists has led to an intriguing and promising use of kiwiberry extracts in the modulation of the immune response in humans and non-human mammals.

## Conclusions

Given the expanding worldwide acceptance and consumption of the kiwiberry, there has been increased interest in identifying, both quantitatively and qualitatively, those health-promoting constituents derived from the fruit. In recent years, there has been a significant increase in the volume of published research on the subject of the kiwiberry’s nutritional value and health benefits. Much of this research points to and confirms a rich composition of health-promoting ingredients. The kiwiberry (*A. arguta*) contains over 20 essential nutrients, making it one of the most nutrient-dense fruits there is. In almost every case, the kiwiberry is more nutrient dense in vitamins, minerals, pigments and phenols than kiwifruit, and is one of the richest sources of both vitamin C and lutein among commonly consumed fruits. The kiwiberry is also considered to be the richest dietary source of *myo*-inositol (vit B_8_). Along with significant amounts of phenolics and essential minerals (primarily potassium, calcium and zinc), it is clear why kiwiberry deserves its status as a ‘health food’ or ‘superfood’. Some of these chemicals (mainly vitamin C and phenolics) possess antioxidant activity. Indeed, considerable research now points to the kiwiberry being a promising natural antioxidant. As such, it could prove helpful in preventing diseases caused by free radicals such as Type I diabetes and certain cardiovascular-related diseases. Early research is already confirming the kiwiberry’s potential in treating hypercholesterolemia, certain cancer types and some gastrointestinal-related diseases.

Additional health-related properties of the kiwiberry, including immunologic benefits, have not yet been fully researched or described, but such research is certainly warranted given this fruit’s growing popularity.

## Electronic supplementary material

ESM 1 (DOCX 1987 kb)

ESM 2 (DOCX 21.8 kb)

ESM 3 (DOCX 17 kb)

## Abbreviations

AA: Antioxidant activity
AAE: Ascorbic acid equivalent
ABTS: 2,2′-azinobis(3-ethylbenzothiazoline-6-sulfonic acid)
CE: Catechin equivalents
CUPRAC: Cupric reducing antioxidant power
DPPH: 2,2-diphenyl-1-picrylhydrazyl
FRAP: Ferric-reducing/antioxidant power
GAE: Gallic acid equivalent
GSH-Px: Glutathione peroxidase
IgE: Immunoglobulin class E
LPS: Lipopolysaccharide
^•^OH: Hydroxyl radical
ORAC: Oxygen radical absorbance capacity
PG102: Water-soluble extract prepared from *Actinidia arguta*
*p*NA: *p*-nitroanilide
RAE: Retinol activity equivalents
SOD: Superoxide dismutase
TAA: Total ascorbic acid content
TDF: Total dietary fibre
TE: Trolox equivalent
TPC: Total phenol content
UPLC-PDA: Ultra performance liquid chromatography – photodiode array detection
