# Supplementary material for: The Nutritional and Health Benefits of Kiwiberry (Actinidia arguta) – a Review
Source: Plant Foods Hum Nutr. 2017 Oct 7;72(4):325–34. doi: 10.1007/s11130-017-0637-y (PMC5717121; doi:10.1007/s11130-017-0637-y)
Supplement: Supplementary file 1 — (DOCX 1987 kb) [file 11130_2017_637_MOESM1_ESM.docx]

The Nutritional and Health Benefits of Kiwiberry (*Actinidia arguta*) – A Review

Plant Foods for Human Nutrition

Piotr Latocha

Department of Environmental Protection, Faculty of Horticulture, Biotechnology and Landscape

Architecture, Warsaw University of Life Sciences – SGGW,

Nowoursynowska 159, 02-776 Warsaw, Poland. E-mail: piotr_latocha@sggw.pl


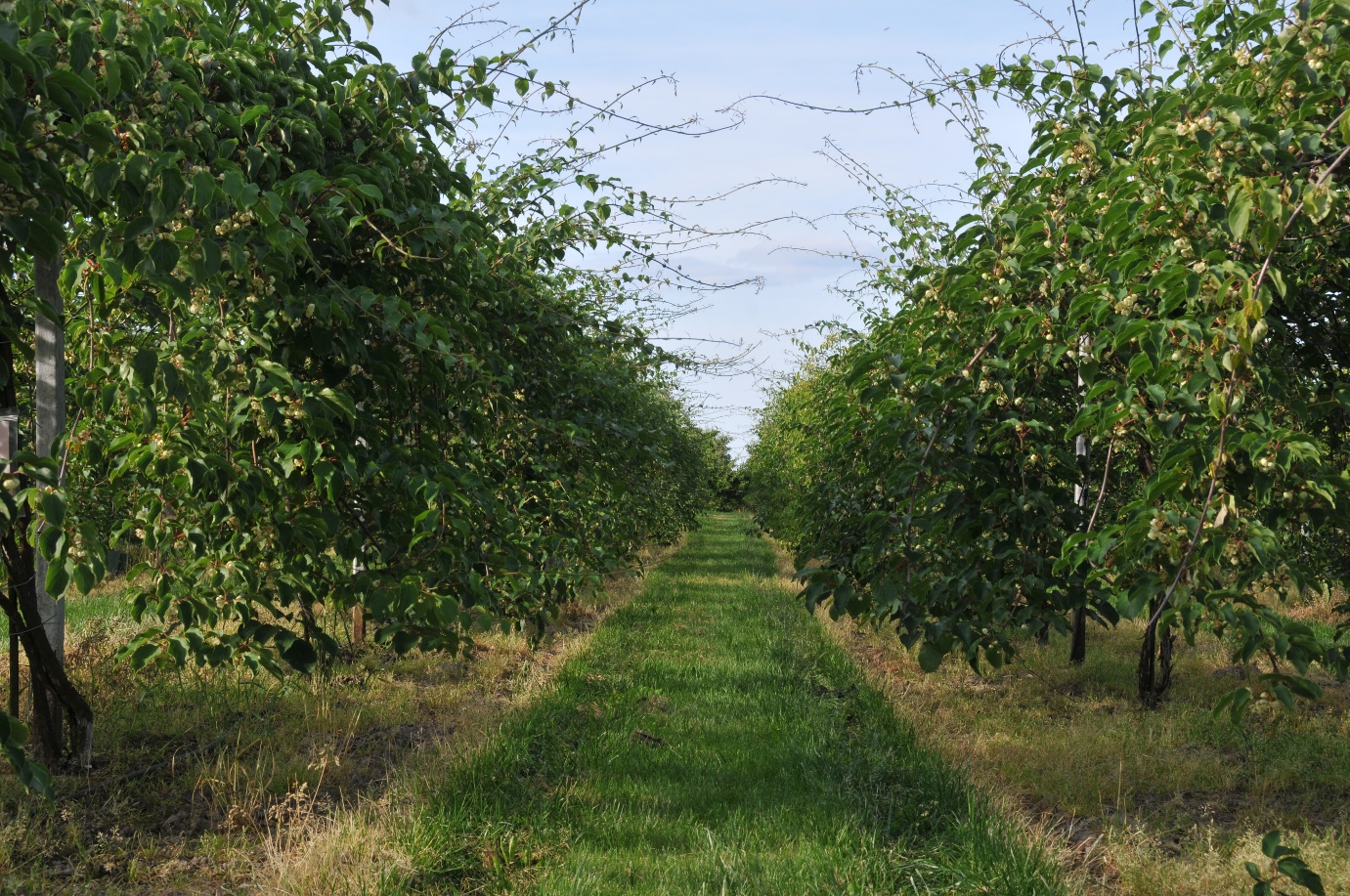


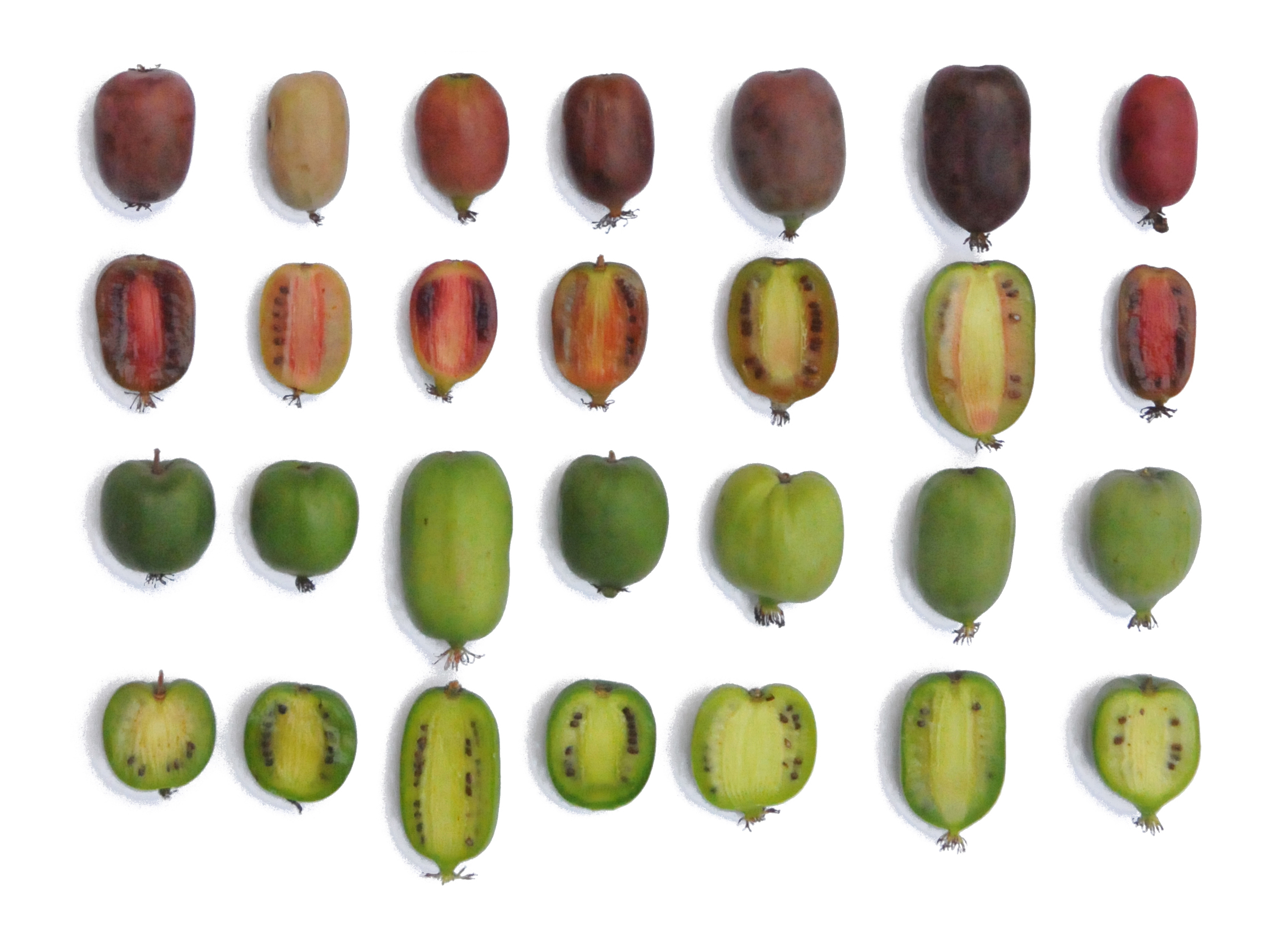


Figure 1. The kiwiberry (*Actinidia arguta*) on commercial plantation and the diversity of its fruit
