# Supplementary material for: The Nutritional and Health Benefits of Kiwiberry (Actinidia arguta) – a Review
Source: Plant Foods Hum Nutr. 2017 Oct 7;72(4):325–34. doi: 10.1007/s11130-017-0637-y (PMC5717121; doi:10.1007/s11130-017-0637-y)
Supplement: Supplementary file 2 — (DOCX 21.8 kb) [file 11130_2017_637_MOESM2_ESM.docx]

The Nutritional and Health Benefits of Kiwiberry (*Actinidia arguta*) – A Review

Plant Foods for Human Nutrition

Piotr Latocha

Department of Environmental Protection, Faculty of Horticulture, Biotechnology and Landscape

Architecture, Warsaw University of Life Sciences – SGGW,

Nowoursynowska 159, 02-776 Warsaw, Poland. E-mail: piotr_latocha@sggw.pl

Table 1. Kiwiberry versus Kiwifruit Nutritional Values of edible portion.

| Nutrient | Units | Kiwiberry  (*Actinidia arguta* different cvs) | Kiwifruit  (*Actinidia*  *deliciosa*  ‘Hayward’) | References |
| --- | --- | --- | --- | --- |
| Dry matter | % | 14.6-25.5 | 15.3-17.2 | 14, 20, 23, 52 |
| Pectin | % | 2.17-3.30 | N/A | 20 |
| Total soluble solid | °Brix | 12.1-24.0 | 13.2-13.8 | 19, 20, 44 |
| Sugars (/100g FW) |  |  |  |  |
| Total soluble sugars | g | 3.9-9.6 | 8.1-8.8 | 20, 24, 37 |
| Sucrose | g | 2.4-7.8 | 0.1-1.6 | 20, 24, 52 |
| Glucose | g | 0.7-3.4 | 3.4-4.1 | 20, 24, 52 |
| Fructose | g | 0.9-2.3 | 3.5-4.4 | 20, 24, 52 |
| Organic acid (/100g FW) |  |  |  |  |
| Total organic acids | g | 1.22-21.06 | 2.19-2.41 | 20, 24 |
| Citric acid | g | 0.54-9.56 | 0.97-1.10 | 20, 24, 52 |
| Quinic acid | g | 0.51-8.20 | 0.78-0.98 | 20, 24, 52 |
| Malic acid | g | 0.10-4.34 | 0.22-0.26 | 20, 24, 52 |
| Oxalic acid | g | 1.70-3.67 | N/A | 20 |
| Shikimic acid | g | 0.03-0.23 | N/A | 20 |
| Succinic acid | g | ND-0.28 | N/A | 20 |
| pH |  | 3.1-3.6 | 3.1-4.0 | 14, 20 |
| Ash | % | 0.62-0.94 | 0.61-0.65 | 14, 20, 52 |
| Total dietary fibre | % FW | 2.0-3.8 | 3.0 | 14, 20, 52 |
|  | % DW | 19.7-27.1 | 12.05-12.13 | 41 |
| Insoluble fibre | % FW | 2.1-3.1 | 2.4 | 14, 20, 52 |
|  | % DW | 14.0-21.5 | 6.8-7.1 | 41 |
| Soluble fibre | % FW | 0.8-1.0 | 0.6 | 14, 20, 52 |
|  | % DW | 5.4-7.1 | 5.0-5.2 | 41 |
| Protease actinidin | mg/mL | 1.6-10.7 | 2.9-4.4 | 30 |
| Protease activity | nM *p*NA/min | 25.7-114.0 | 6.3-10.1 | 30 |
| Vitamins (/100g FW) |  |  |  |  |
| Vitamin C (Total ascorbic acid) | mg | 22.8-430 | 61.8-116.6 | 17,19, 20, 32, 31, 40 |
| Vitamin B_1_ (Thiamine) | mg | 0.01-0.05 | ND-0.03 | 14, 35, 52 |
| Vitamin B_2_ (Riboflavin) | mg | 0.02-0.11 | 0.03-0.05 | 14, 35, 36, 52 |
| Vitamin B_3_ (Niacin) | mg | 0.50-1.55 | 0.34-0.83 | 14, 37, 52 |
| Vitamin B_5_ (Pantothenic acid) | mg | 3.80-5.60 | ND-0.18 | 14, 37, 52 |
| Vitamin B_6_ (Pyridoxine) | mg | 1.10-1.90 | 0.06-0.07 | 14, 37, 52 |
| Vitamin B_8_ (*Myo*-inositol) | mg | 266-982 | 97-135 | 24, 40 |
| Vitamin B_9_ (Folic acid) | µg | N/A | 25.0-38.2 | 14, 52 |
| Vitamin E (*α*-tocopherol) | mg | 4.60-5.28 | 0.86-1.46 | 14, 36, 52 |
| Vitamin K (Phylloquinone) | µg | N/A | 11.0-40.3 | 14, 52 |
| Vitamin A (RAE) | µg | 37.7-84.5 | 4.0-9.02 | 14, 40, 52 |
| Phenolic compounds (/100g FW) |  |  |  |  |
| Total polyphenols (GAE) | mg | 79.0-426.7 | 41.7-267.0 | 14, 19, 21, 43-45 |
| Total phenolic compounds  (HPLC, as sum of identified compounds) | mg | 443.2-1301.1 | N/A | 20 |
| Total flavonoids (CE) | mg | 28.8-87.2 | 67.6 | 21, 43 |
| Total flavonols  (HPLC as sum of identified compounds) | mg | 1.7-18.9 | N/A | 20 |
| Flavan-3-ols (as polymeric procyanidins, HPLC) | mg | 440-1282 | N/A | 20 |
| Tannins (CE) | mg/g DW | 2.3-9.3 | 1.8-2.0 | 41 |
| Total phenolic acids | mg | 1.6-8.1 | N/A | 20 |
| Carotenoids (/100g FW) |  |  |  |  |
| β-carotene | mg | 0.22-0.29 | 0.05-0.07 | 14, 17, 46 |
| *α*-carotene | mg | ND-0.09 | ND | 17, 52 |
| Lutein | mg | 0.26-0.93 | 0.11-0.42 | 46 |
| Zeaxanthin | mg | 0.02-0.04 | ND | 17, 52 |
| Violaxanthin | mg | 0.01-0.12 | ND | 17, 52 |
| Chlorophylls (*a* + *b*) | mg/100g FW | 2.6-4.2 | 1.3-2.7 | 17, 46 |
| Anthocyanins | µg/100g FW | 161.2-206.1 | ND | 47 |
|  | µg/g DW | ND-129.8 | ND | 20, 41 |
| Macroelements (/100g FW) |  |  |  | 14, 16, 35, 37, 50-52 |
| Potassium (K) | mg | 163-382 | 301-312 |  |
| Calcium (Ca) | mg | 51.5-120.1 | 27.0-34.0 |  |
| Phosphorous (P) | mg | 31.7-80.2 | 33.8-34.0 |  |
| Magnesium (Mg) | mg | 10.0-23.2 | 14.4-17.0 |  |
| Sodium (Na) | mg | 1.2-9.6 | 2.3-3.0 |  |
| Microelements (/100g FW) |  |  |  | 14, 16, 35, 37, 50, 52 |
| Iron (Fe) | mg | 0.31-1.15 | 0.22-0.31 |  |
| Zinc (Zn) | mg | 0.18-1.45 | 0.10-0.14 |  |
| Copper (Cu) | mg | 0.05-0.16 | 0.12-0.13 |  |
| Manganese (Mn) | mg | 0.03-0.24 | 0.08-0.10 |  |
| Selenium (Se) | µg | N/A | 0.20-0.60 |  |
| Boron (B) | mg | 0.18-0.48 | N/A |  |
| Total amino acids (/100g FW) | mg | 601-1220 | 875-1058 | 14, 37, 52 |
| Isoleucine | mg | 28.3-65.1 | 40.0-51.0 | 14, 37, 52 |
| Histidine | mg | 13.9-25.5 | 17.0-27.0 | 14, 37, 52 |
| Leucine | mg | 44.3-74.6 | 55.0-66.0 | 14, 37, 52 |
| Lysine | mg | 32.4-67.3 | 51.0-61.0 | 14, 37, 52 |
| Methionine | mg | 8.2-8.6 | 17.0-24.0 | 14, 37, 52 |
| Phenylalanine | mg | 25.7-56.4 | 33.0-44.0 | 14, 37, 52 |
| Threonine | mg | 28.7-74.0 | 44.0-47.0 | 14, 37, 52 |
| Valine | mg | 30.9-67.5 | 46.0-57.0 | 14, 37, 52 |
| Cysteine | mg | 8.0-21.5 | 23.0-31.0 | 14, 37, 52 |
| Arginine | mg | 42.6-70.1 | 59.0-81.0 | 14, 37, 52 |
| Aspartic acid | mg | 75.7-139.8 | 111.0-126.0 | 14, 37, 52 |
| Glutamic acid | mg | 96.4-190.3 | 138.0-184.0 | 14, 37, 52 |
| Alanine | mg | 53.3-101.2 | 44.0-53.0 | 14, 37, 52 |
| Glycine | mg | 34.1-82.8 | 48.0-60.0 | 14, 37, 52 |
| Proline | mg | 26.6-50.2 | 36.0-44.0 | 14, 37, 52 |
| Serine | mg | 30.6-63.6 | 35.0-53.0 | 14, 37, 52 |
| Tyrosine | mg | 21.4-62.5 | 29.0-34.0 | 14, 37, 52 |

Abbreviations: N/A – Data not available; DW- dry weight; FW-fresh weight; RAE - Retinol activity equivalent; ND – Not detected; CE, Catechine equivalent; GAE, Gallic acid equivalent; *p*NA, *p*- nitroanilide
