# Supplementary material for: The Nutritional and Health Benefits of Kiwiberry (Actinidia arguta) – a Review
Source: Plant Foods Hum Nutr. 2017 Oct 7;72(4):325–34. doi: 10.1007/s11130-017-0637-y (PMC5717121; doi:10.1007/s11130-017-0637-y)
Supplement: Supplementary file 3 — (DOCX 17 kb) [file 11130_2017_637_MOESM3_ESM.docx]

The Nutritional and Health Benefits of Kiwiberry (*Actinidia arguta*) – A Review

Plant Foods for Human Nutrition

Piotr Latocha

Department of Environmental Protection, Faculty of Horticulture, Biotechnology and Landscape

Architecture, Warsaw University of Life Sciences – SGGW,

Nowoursynowska 159, 02-776 Warsaw, Poland. E-mail: piotr_latocha@sggw.pl

Table 2. The differences in kiwiberry and kiwifruit antioxidant capacity based on different methods.

| Assay | Units | Kiwiberry  (*Actinidia arguta* different cvs) | Kiwifruit (*Actinidia deliciosa* ‘Hayward’) | References |
| --- | --- | --- | --- | --- |
| ABTS^1^ | µM TE/g DW | 47.2-122.4 | 13.5-16.1 | 41 |
| ABTS^1^ | mg AAE/g FW | 1.73-4.22 | 1.83-2.06 | 17, Latocha unpubl. |
|  |  | 1.10-2.10 | N/A | 43 |
| ABTS^2^ | µM TE/g DW | 10.8-29.9 | 11.1-13.5 | 41 |
| ABTS^2^ | mg AAE/g FW | 0.93-2.64 | 0.95-1.36 | 17, 54, Latocha unpubl. |
| DPPH^1^ | µM TE/g DW | 10.5-42.5 | 6.6-7.9 | 41 |
| DPPH^2^ | µM TE/g DW | 9.0-15.7 | 6.0-6.9 | 41 |
| DPPH^2^ | mg AAE/g FW | 0.99-2.64 | 0.97-0.99 | 17, Latocha unpubl. |
|  |  | 0.81-2.90 | N/A | 43 |
|  |  | 1.7-1.9 | N/A | 44 |
| CUPRAC^1^ | µM TE/g DW | 31.9-104.5 | 11.5-23.6 | 41 |
| FRAP^1^ | µM TE/g DW | 10.7-24.5 | 7.8-9.4 | 41 |
| FRAP^2^ | µM TE/g DW | 9.3-15.1 | 8.2-10.1 | 41 |
| ORAC^1^ | µM TE/g FW | 88.7-99.7 | 5.2-7.1 | 45 |
| ORAC^1^ | mg AAE/g FW | 8.8-13.9 | N/A | 43 |
| ORAC^2^ | µM TE/g FW | 0.4-0.7 | 0.3-0.5 | 45 |
| ^•^OH ^1^ | % | 50.4-70.7 | 57.5-62.2 | 17 |

^1^ Hydrophilic assay; ^2^ Lipophilic assay

Abbreviations: ABTS, 2,2-azinobis-(3-ethylbenzthiazoline-6-sulfonic acid); DPPH, 1,1-diphenyl-2-picrylhydrazyl; CUPRAC, Cupric reducing antioxidant capacity; FRAP, Ferric-reducing/antioxidant power; ORAC, Oxygen Radical Absorbance Capacity ^•^OH^,^ Hydroxyl radical; TE, Trolox equivalent; AAE, Ascorbic acid equivalent; FW, fresh weight; DW, dry weight, N/A – Data not available
